# Analysis of clinical and imaging features and prognosis of patients positive for *Tropheryma whipplei* detected by nanopore sequencing of bronchoalveolar lavage fluid

**DOI:** 10.3389/fcimb.2025.1652182

**Published:** 2025-09-30

**Authors:** Chang Song, Chun-Yan Zhao, Mei Yu, Chang-Yue Jiang, Hang-Biao Qiang, Ren-Hao Liu, Xiao-Mei Yang, Zhou-Hua Xie, Qing-Dong Zhu

**Affiliations:** ^1^ Department of Pharmacy, Fourth People's Hospital of Nanning, Nanning, Guangxi, China; ^2^ Clinical Medical School, Guangxi Medical University, Nanning, Guangxi, China; ^3^ Department of Anesthesiology, The Fourth People’s Hospital of Nanning, Nanning, Guangxi, China; ^4^ School of Basic Medical Science, Guangxi Medical University, Nanning, Guangxi, China

**Keywords:** bronchoalveolar lavage fluid, nanopore sequencing, *Tropheryma whipplei*, clinical features, prognosis

## Abstract

**Background:**

To probe into the application value of nanopore sequencing in patients suffering from positive *Tropheryma whipplei* (TW), analyze their clinical features, imaging manifestations, and treatment prognosis, and provide new evidence for the diagnosis and treatment of Whipple’s disease.

**Methods:**

This study retrospectively analyzed 2,137 samples subjected to nanopore sequencing at the Fourth People’s Hospital of Nanning. Among them, 14 bronchoalveolar lavage fluid (BALF) samples were positive for TW. Patients were divided into a high-sequence group (100) and a low-sequence group (≤100) in accordance with the TW sequence counts. The clinical features, laboratory indicators, imaging manifestations, and treatment prognosis of these two groups were compared and analyzed in an all-round manner.

**Results:**

The levels of C-reactive protein (CRP) and lactate dehydrogenase (LDH) in the high-sequence group were strikingly higher than those in the low-sequence group (P < 0.05). Radiologically, TW pneumonia mostly presented as patchy shadows, ground-glass opacities, and bronchiectasis, which highly overlapped with the imaging features of tuberculosis or interstitial pneumonia. With respect to treatment, 85.7% of the 14 patients exhibited symptomatic improvement subsequent to antibiotic treatment. Fluoroquinolones and combination therapy regimens demonstrated satisfactory efficacy, but patients suffering from severe conditions or advanced age may require an extended treatment course.

**Conclusion:**

The imaging manifestations of TW pneumonia are not only non-specific but also have been frequently mistaken with other pulmonary diseases. Notwithstanding the fact that fluoroquinolones and combination therapy regimens hold clinical relevance in the management of Whipple’s disease, tailored adjustments to treatment plans are crucial based on individual patient circumstances.

## 1 Introduction

Whipple’s disease, an uncommon systemic infectious ailment induced by *Tropheryma whipplei* (TW), was initially delineated by George H. Whipple in 1907. Predominantly targeting the small intestine, this condition has the potential to infiltrate various other organ systems, encompassing the heart, lungs, joints, and central nervous system (Lin et al., 2022; Cappellini et al., 2024). The clinical manifestations of Whipple’s disease are diverse, ranging from digestive system symptoms such as diarrhea, abdominal pain, and weight loss to joint pain, fever, and neurological symptoms (Holstein et al., 2023). On account of its rarity and non-specific clinical manifestations, Whipple’s disease frequently escapes initial diagnosis or is misidentified, resulting in treatment delays and adverse patient outcomes (Motwade and Osman Taha, 2023). Nonetheless, the existing diagnostic criteria mainly hinge significantly on the histopathological finding of periodic acid-Schiff (PAS) stain-positive macrophage infiltration, and its sensitivity strikingly decreases in non-intestinal samples.

In recent years, advancements in molecular biology techniques, particularly the integration of 16S rRNA gene sequencing and polymerase chain reaction (PCR) methodologies (Van Dong et al., 2025), have significantly enhanced the diagnostic precision of Whipple’s disease (Meyer et al., 2023). Nevertheless, these techniques may encounter multiple constraints in specific scenarios, such as sample quality, DNA extraction efficiency, and PCR inhibitors. Nanopore sequencing technology stands as an emerging high-throughput sequencing approach. As a consequence of its rapidity, simplicity, and high sensitivity, it presents enormous potential in the diagnosis of infectious diseases (Girgis et al., 2023; de Cesare et al., 2024). In comparison with traditional sequencing technologies, nanopore sequencing directly sequences the DNA or RNA in the sample without PCR amplification, thus avoiding the biases and errors that may be introduced throughout the PCR amplification process (Song et al., 2024a). Its single-molecule resolution can effectively identify low-abundance microbial signals and achieve high-precision species identification through the dynamic time correction algorithm. In the realm of clinical practice, bronchoalveolar lavage fluid (BALF) is a liquid collected after lung lavage via bronchoscopy. Originating directly from the affected lung area and typically containing a high concentration of pathogen DNA, BALF serves as a pivotal sample type for such diagnoses (Hogea et al., 2020). As a primary systemic infection targeting the small intestine, Whipple’s disease infrequently involves the lungs. Nevertheless, in certain instances, pulmonary symptoms may dominate the clinical manifestations of the disease. These symptoms may include persistent cough, dyspnea, chest pain, and pulmonary infiltration, which may occur alone or coexist with symptoms of other systems (Ladna et al., 2024). Furthermore, diagnosis is often challenging attributable to the non-specific nature demonstrated within the pulmonary symptoms of Whipple’s disease and their similarity to other respiratory diseases. Aside from that, individuals suffering from Whipple’s disease with pulmonary involvement may exhibit atypical features in imaging examinations, such as nodules, consolidation, or interstitial pneumonia, which further heightens the complexity of diagnosis (Hofmann et al., 2021). On that account, nanopore sequencing detection by employing BALF samples may improve early diagnosis and treatment for individuals enduring pulmonary symptoms, especially those whose etiology cannot be clearly identified by conventional diagnostic methods.

Nevertheless, to our knowledge, there is a notable lack of research exploring the utilization of this technology in diagnosing Whipple’s disease. On that account, this study endeavors to delve into the application value of nanopore sequencing technology in diagnosing Whipple’s disease by comprehensively analyzing the clinical manifestations, imaging characteristics, and treatment outcomes of 14 patients who tested positive for TW using nanopore sequencing. We hope that this study can furnish clinicians with insightful references and contribute to novel perspectives and approaches for the diagnosis and management of Whipple’s disease, ultimately enhancing patient care and medical services.

## 2 Materials and methods

### 2.1 Sample collection

Prior to sample collection, patients must be thoroughly informed and adequately prepared, with a fasting period of six hours preceding the procedure to guarantee their safety. Local anesthesia and sedative drugs were used throughout the operation to relieve discomfort, and bronchoalveolar lavage was performed by inserting a bronchoscope into the target bronchus. After ensuring that the tip of the bronchoscope was accurately located in the required pulmonary segment, 50–100 milliliters of sterile normal saline was used for each lavage. In accordance with the patient’s tolerance and clinical needs, the lavage was repeated 3 to 5 times. After each lavage, negative pressure suction of 25 to 100 mmHg was applied, and the bronchoalveolar lavage fluid (BALF) was recovered by adopting the “point suction” technique. This method not only minimizes the collapse of the bronchial lumen, but also augments the recovery amount, ensuring that the total recovery rate exceeds 30%.

In an effort to guarantee the depth and comprehensiveness of our research, we have strictly designed and implemented quality control measures at every stage of this study. Throughout the sample collection phase, the protocols for bronchoalveolar lavage fluid (BALF) collection and nanopore sequencing were referenced from The Official American Thoracic Society Clinical Practice Guideline (Meyer et al., 2012) and Instruction for the Application of Nanopore Sequencing Technology (Wang et al., 2021). In this study, healthcare personnel involved in sample collection received comprehensive and systematic standardization training. This specifically covered the standardized operating procedures for sample collection, key points for attention, and methods for dealing with unexpected situations. For the collected samples, strict transportation and storage protocols were established, with proper handling in accordance with the specified temperature, time, and other requirements.

### 2.2 Nanopore sequencing

In this study, sample pretreatment was performed in a biosafety cabinet. The operation table was disinfected with 84 disinfectant, 75% alcohol, and UV irradiation for 30 minutes. Samples were ground in EP tubes with DTT solution, proteinase K, lysozyme, and zirconia beads using a grinder. Nucleic acids were extracted using a third-generation nanopore sequencing kit and quality-controlled with Qubit 4.0. The PCR reaction (30 μL) contained 2×PCR Mix, primers, and template, and was purified with magnetic beads. Purified DNA was barcoded, used to construct a library, and 100 ng was sequenced. Sequencing data were collected via GridION and MinKNOW, filtered to remove fragments <200 bp and host DNA, and aligned with pathogen and drug resistance databases. All steps strictly followed kit instructions and guidelines to ensure accuracy (**Supplementary Material 1**).

### 2.3 Positive report criteria and patient grouping

By utilizing the detection of nucleic acids in samples as its foundation, this study employs a detection method integrating targeted amplification with metagenomic sequencing. The coverage of metagenomic sequencing encompasses an extensive range of 26,260 microbial species such as bacteria, fungi, viruses, and atypical pathogens. In developing criteria for interpreting positive nanopore sequencing results, this research draws upon a comprehensive suite of indicators, integrating insights from prior studies (Gu et al., 2021; Lai et al., 2024) and the Expert consensus on clinical standardized application of metagenomics next‐generation sequencing for detection of pathogenic microorganisms (Zhang et al., 2024).

In accordance with the final number of detected TW sequences, patients were categorized into two distinct groups: a high-sequence cohort (100 sequences) and a low-sequence cohort (≤100 sequences). Subsequently, a detailed comparative analysis was carried out on the clinical characteristics and laboratory test indicators of these two groups of patients. This categorization approach is intended to delve into the potential association between the TW sequence counts as well as the clinical manifestations and laboratory test results of patients. By comparing the clinical symptoms, signs, laboratory test results, and imaging features of these two groups of patients, we expect to elucidate the influence of different TW sequence levels on disease manifestation and progression.

### 2.4 Collection of clinical information of positive patients and ethical approval

This investigation conducted a retrospective analysis on 2,137 samples subjected to nanopore sequencing at the Fourth People’s Hospital of Nanning from July 2021 to January 2025. Notably, 14 samples tested positive for TW. For these 14 patients with positive results, we leveraged the electronic medical record system to gather comprehensive clinical data, encompassing age, gender, race, past medical history, current laboratory tests, drug use, diagnosis and treatment process, and prognosis follow-up. Ethical approval was granted by the Ethics Committee of the Fourth People’s Hospital of Nanning (Approval No: [2023] 24). This retrospective study does not contain any identifiable human images and employs an anonymous data collection method, thereby underscoring the necessity for written informed consent from patients. Afterwards, all information that could be used to identify patients (such as names, addresses, etc.) has been removed throughout the data collection phase. Moreover, all data are presented in an anonymized form during aggregation and analysis. Subsequently, the correspondence between codes and patient identities is only accessible by authorized personnel when necessary. The study strictly adheres to the Declaration of Helsinki, and all patient data have been anonymized and handled with strict confidentiality to protect patient privacy.

### 2.5 Collection of imaging materials

In this investigation, we employed a state-of-the-art GE LightSpeed 64-slice spiral CT scanner to obtain imaging data. Patients were initially instructed to lie flat on the scanning bed and subsequently advised to remain still throughout the scanning procedure, refraining from any activities that might give rise to motion artifacts. All CT scans were performed from the apex to the root of the lung to ensure coverage of the entire lung area. The scanning reconstruction parameters were meticulously configured as below: a tube voltage of 120 kV, an effective tube power of 30 mAs, a detector collimation of 128×0.625 mm, and a matrix resolution of 512×512. Without injecting contrast agents, the scanning slice thickness was set at 5 mm, and the CDTIvol value was 2.03 mGy to obtain clear lung images. Subsequently, the entire sequence of the obtained images was exported, and professional radiologists determined the most typical lung lesion images for display.

### 2.6 Statistical analysis

A statistical investigation was conducted by adopting IBM SPSS Statistics v23.0, Armonk, NY and GraphPad Prism v9.0, San Diego, CA. Categorical variables were described and investigated by utilizing frequencies and constituent ratios, while continuous variables were expressed as mean ± standard deviation. In the intergroup comparison, the Mann-Whitney U test was chosen for analysis based on the distribution characteristics of continuous variables, while the difference in the composition of categorical variables was analyzed by carrying out Fisher’s exact test.

## 3 Results

### 3.1 Clinical baseline characteristics of the included patients

This study retrospectively analyzed 2,137 samples subjected to nanopore sequencing at the Fourth People’s Hospital of Nanning from July 2021 to January 2025, among which 14 bronchoalveolar lavage fluid (BALF) samples were detected to be positive for TW. On the basis of the sample grouping principles and indications from previous studies (Chen et al., 2022), patients were divided into two groups in line with their sequence numbers: the high-sequence group (100) (n = 8) and the low-sequence group (≤100) (n = 6). The hypothesis setting for the group comparison is as follows: H_0_: There is no conspicuous distinction between these two groups in clinical characteristics, blood indicators, etc.; H_1_: There is a remarkable disparity between these two groups in clinical characteristics, blood indicators, etc. If P < 0.05, H_0_ is rejected, and it is concluded that there is no noticeable discrepancy between these two groups in clinical characteristics, blood indicators, etc. If P > 0.05, the conclusion is the opposite. As suggested by the results displayed in **Table 1**, there were no significant differences in gender and age distribution between these two groups. With respect to clinical manifestations, the high-sequence group exhibited higher proportions of fever, cough, and expectoration compared to the low-sequence group. Nonetheless, the discrepancies failed to attain statistical significance. With respect to comorbidities, the proportion of individuals suffering from a history of tuberculosis in the high-sequence group was higher than that in the low-sequence group. By comparison, the proportion of diabetes was lower than that in the low-sequence group, but these differences were also not statistically significant. Furthermore, no remarkable discrepancies existed in the distribution of other comorbidities such as hepatitis B, hypertension, coronary heart disease, etc. between these two groups. As the above research findings demonstrate, there may be a certain association between the TW sequence counts and the clinical characteristics of patients, but more in-depth research is essential for verification.

**Table 1:** Clinical baseline characteristics of *Tropheryma Whipplei*-positive patients.

Characteristics	Low sequence group (n=6)	High sequence group (n=8)	Total	P value
Gender (Male, Female)	4,2	7,1	11,3	0.538
Age (x ± s)	39.00 ± 18.10	43.88 ± 19.33	41.79 ± 18.26	0.755
Clinical Manifestations				
Fever	0(0/6, 0.00%)	1(1/8, 12.50%)	1(1/14, 7.14%)	1.000
Cough	2(2/6, 33.33%)	6(6/8, 75.00%)	8(8/14, 57.14%)	0.277
Sputum production	2(2/6, 33.33%)	6(6/8, 75.00%)	8(8/14, 57.14%)	0.277
Fatigue	0(0/6, 0.00%)	1(1/8, 12.50%)	1(1/14, 7.14%)	1.000
Abdominal pain	0(0/6, 0.00%)	1(1/8, 12.50%)	1(1/14, 7.14%)	1.000
Abdominal distension	0(0/6, 0.00%)	1(1/8, 12.50%)	1(1/14, 7.14%)	1.000
Ascites	0(0/6, 0.00%)	1(1/8, 12.50%)	1(1/14, 7.14%)	1.000
Pulmonary rales	0(0/6, 0.00%)	1(1/8, 12.50%)	1(1/14, 7.14%)	1.000
Comorbidities				
Tuberculosis	2(2/6, 33.33%)	5(5/8, 62.50%)	7(7/14, 50.00%)	0.592
Diabetes mellitus	2(2/6, 33.33%)	0(0/8, 0.00%)	2(2/14, 14.29%)	0.165
Hepatitis B	1(1/6, 16.67%)	1(1/8, 12.50%)	2(2/14, 14.29%)	1.000
Hypertension	1(1/6, 16.67%)	1(1/8, 12.50%)	2(2/14, 14.29%)	1.000
Coronary heart disease	1(1/6, 16.67%)	0(0/8, 0.00%)	1(1/14, 7.14%)	0.429
Cerebral infarction	0(0/6, 0.00%)	1(1/8, 12.50%)	1(1/14, 7.14%)	1.000
Rheumatoid arthritis	1(1/6, 16.67%)	0(0/8, 0.00%)	1(1/14, 7.14%)	0.429
Renal insufficiency	1(1/6, 16.67%)	1(1/8, 12.50%)	2(2/14, 14.29%)	1.000
Gastrointestinal inflammation	1(1/6, 16.67%)	1(1/8, 12.50%)	2(2/14, 14.29%)	1.000

### 3.2 Laboratory tests

**Figure 1:**
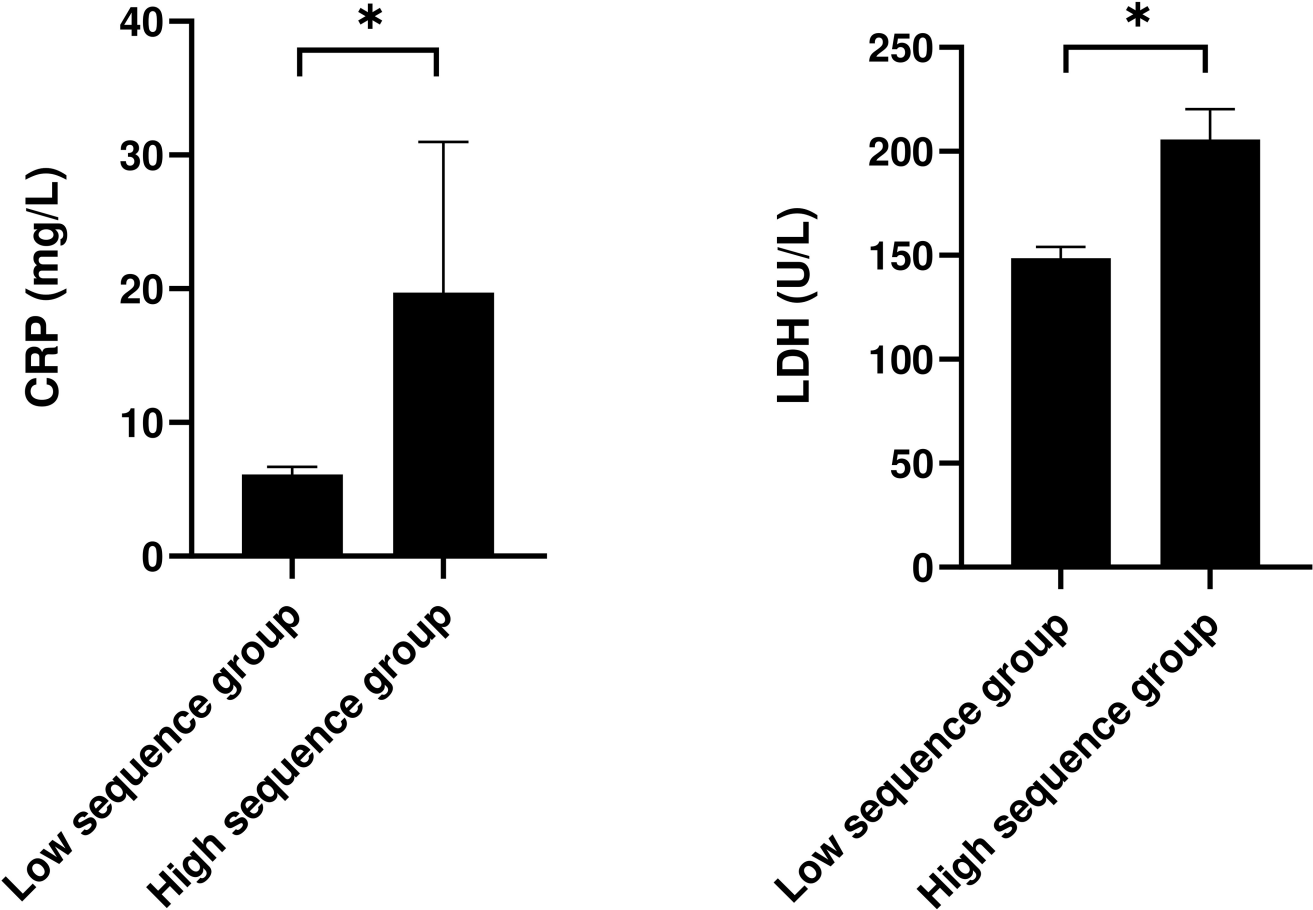
Characteristic laboratory tests of patients in different groups. *:P<0.05.

This study compared and analyzed the laboratory indicators of patients stratified into the low-sequence group and the high-sequence group (**Table 2**). The findings revealed no statistically discernible differences between these two groups across a spectrum of indicators, encompassing white blood cell count, red blood cell count, hemoglobin level, platelet count, neutrophils, lymphocytes, monocytes, eosinophils, basophils, alanine aminotransferase (ALT), aspartate aminotransferase (AST), total protein (TP), albumin (ALB), urea (Scr), potassium ion (K+), sodium ion (Na+), etc. (P > 0.05). Nonetheless, the C-reactive protein (CRP) level in the high-sequence group was dramatically higher than that in the low-sequence group (27.13 ± 31.21 vs 6.12 ± 1.36, P = 0.034), and the lactate dehydrogenase (LDH) level was also dramatically higher than that in the low-sequence group (205.83 ± 40.87 vs 148.73 ± 13.24, P = 0.021). Apart from that, although the CD4+/CD8+ T-cell ratio was marginally increased in the high-sequence group relative to the low-sequence group, this difference failed to attain statistical significance (P = 0.054) (**Figure 1**).

**Table 2:** Laboratory examination.

Laboratory indicators	Low sequence group	High sequence group	Total	P value
White blood cells	6.07 ± 2.54	7.61 ± 1.34	6.95 ± 2.02	0.108
Red blood cells	4.90 ± 0.61	4.51 ± 0.50	4.68 ± 0.57	0.282
Hemoglobin	131.50 ± 26.24	128.88 ± 25.86	130.00 ± 25.04	0.755
Platelets	319.67 ± 68.47	342.63 ± 138.78	332.79 ± 110.96	0.662
Neutrophils	4.08 ± 2.21	5.23 ± 1.05	4.74 ± 1.68	0.181
Lymphocytes	1.63 ± 0.54	1.69 ± 0.59	1.66 ± 0.55	0.852
Monocytes	0.50 ± 0.23	0.55 ± 0.16	0.53 ± 0.19	0.491
Eosinophils	0.17 ± 0.08	0.21 ± 0.11	0.19 ± 0.10	0.414
Basophils	0.04 ± 0.01	0.04 ± 0.01	0.04 ± 0.01	0.662
CRP	6.12 ± 1.36	27.13 ± 31.21	18.12 ± 25.33	0.005
ESR	41.00 ± 33.24	50.00 ± 30.27	46.14 ± 30.66	0.414
ALT	14.38 ± 3.52	17.90 ± 4.87	16.39 ± 4.56	0.228
AST	18.47 ± 10.30	17.85 ± 20.91	18.11 ± 16.62	0.414
TP	70.53 ± 6.26	70.95 ± 5.59	70.77 ± 5.65	0.852
ALB	43.33 ± 2.22	42.49 ± 6.74	42.85 ± 5.15	0.755
LDH	148.73 ± 13.24	205.83 ± 40.87	181.36 ± 42.74	0.001
Scr	66.67 ± 18.16	67.38 ± 14.14	67.07 ± 15.32	0.755
K+	3.67 ± 0.39	3.78 ± 0.23	3.74 ± 0.30	0.228
Na+	140.38 ± 1.36	138.19 ± 2.08	139.13 ± 2.08	0.290
CD3+	979.83 ± 255.33	1154.63 ± 608.24	1079.71 ± 482.01	0.755
CD4+	540.83 ± 113.95	654.75 ± 284.87	605.93 ± 228.28	0.345
CD8+	384.50 ± 129.27	424.38 ± 291.53	407.29 ± 229.37	1
CD4+/CD8+	1.49 ± 0.35	1.82 ± 0.67	1.68 ± 0.56	0.573

### 3.3 Imaging features

**Figure 2:**
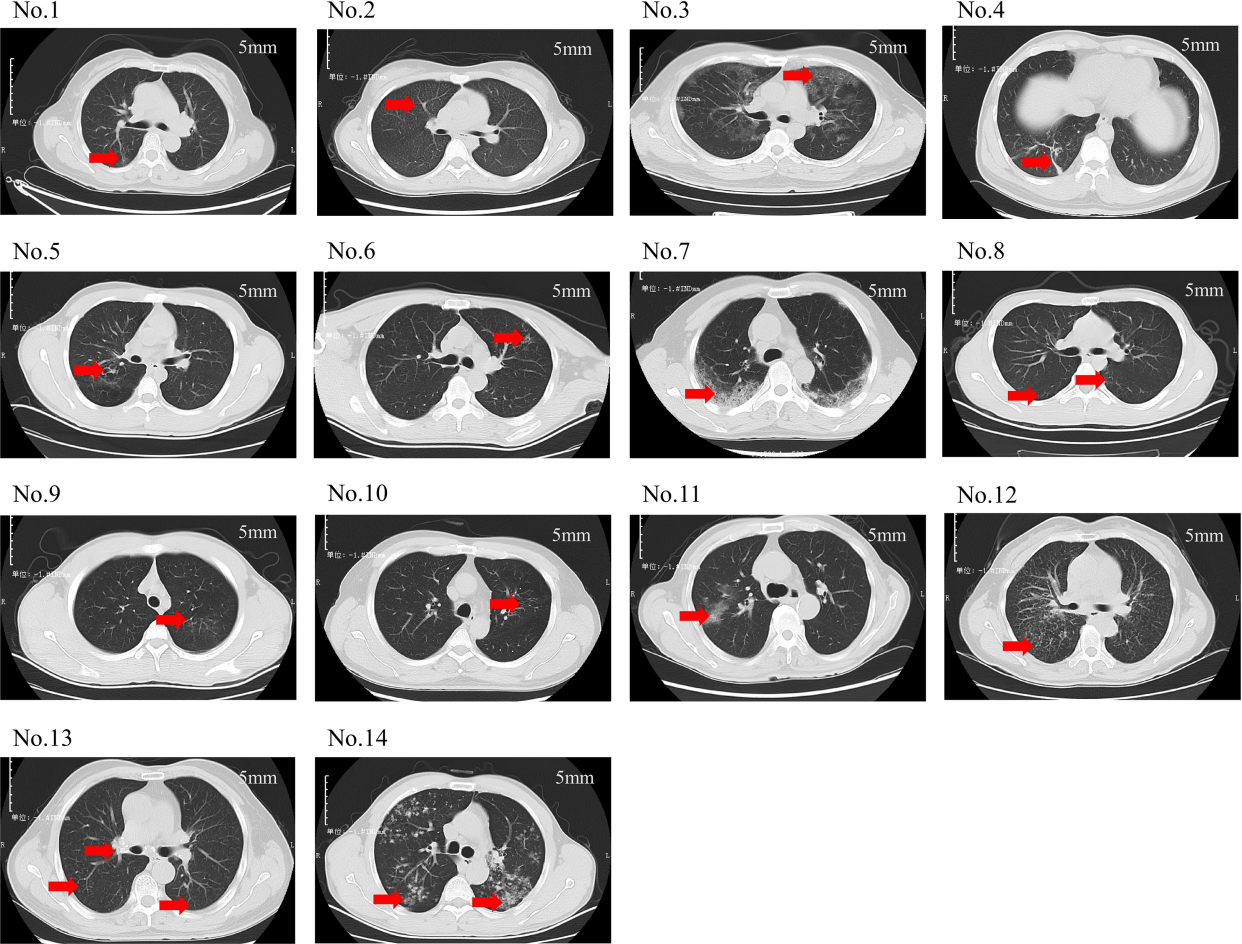
Chest CT scans of 14 patients. (No.1: Linear opacities are observed in the posterior segment of the right upper lobe. No.2: Diffuse miliary nodules are distributed throughout both lungs. No.3: Ground-glass opacities are noted in all lobes of both lungs. No.4: Scattered patchy and nodular shadows are observed in the posterior basal segment of the right lower lobe. No.5: Bronchiectasis is identified in the posterior segment of the right upper lobe. No.6: A small cavity is formed in the left upper lobe. No.7: Spotty, patchy, and linear shadows are seen near the pleura in both lungs. No.8: Small nodular shadows are observed in the dorsal segment of both lower lobes. No.9: Minor ground-glass opacities are noted in the apical posterior segment of the left upper lobe. No.10: Scattered spotty and linear opacities are present in the apical posterior segment of the left upper lobe. No.11: Patchy shadows are observed in the right upper lobe. No.12: Multiple miliary nodules are distributed throughout both lungs. No.13: Scattered miliary nodules are noted in both lungs, accompanied by calcification of the hilar lymph nodes. No.14: Multiple nodules and patchy shadows are observed in both lungs, with a cavity identified in the dorsal segment of the left lower lobe. This translation adheres to the formal and precise language typically used in academic papers).

**Figure 2** depicts the pulmonary imaging manifestations of 14 positive patients. A further comparative analysis on the imaging features of patients was carried out in the low-sequence group and the high-sequence group (**Table 3**). As our investigation findings illustrated, a total of 13 patients exhibited patchy shadows. Additionally, the occurrence rates in the low-sequence group and the high-sequence group were 100.00% and 87.50%, respectively. Cord-like shadows were observed in 11 patients, and the occurrence rates in these two groups were 83.33% and 75.00%, respectively. Bronchiectasis occurred in 5 patients, and the occurrence rates in these two groups were 33.33% and 37.50%, respectively. Nodules occurred in 7 patients, and the occurrence rates in both groups were 50.00%. Cavitation was merely observed in 1 patient, exclusively in the high-sequence group. Ground-glass opacities were present in 2 patients, both belonging to the low-sequence group. Rare manifestations included pericardial effusion and enlargement of mediastinal and hilar lymph nodes. No statistically significant differences were noted in the distribution of these imaging features between the two patient groups.

**Table 3:** Imaging characteristics of patients in these two groups.

Radiological features	Low sequence group	High sequence group	Total	P value
Patchy opacity	6(6/6, 100.00%)	7(7/8, 87.50%)	13(13/14, 92.86%)	1.000
Linear opacity	5(5/6, 83.33%)	6(6/8, 75.00%)	11(11/14, 78.57%)	1.000
Bronchiectasis	2(2/6, 33.33%)	3(3/8, 37.50%)	5(5/14, 35.71%)	1.000
Nodule	3(3/6, 50.00%)	4(4/8, 50.00%)	7(7/14, 50.00%)	1.000
Cavity	0(0/6, 0.00%)	1(1/8, 12.50%)	1(1/14, 7.14%)	1.000
Ground-glass opacity	2(2/6, 33.33%)	0(0/8, 0.00%)	2(2/14, 14.29%)	0.165
Pericardial effusion	1(1/6, 16.67%)	0(0/8, 0.00%)	1(1/14, 7.14%)	0.429
Mediastinal and hilar lymphadenopathy	1(1/6, 16.67%)	1(1/8, 12.50%)	2(2/14, 14.29%)	1.000

### 3.4 Treatment regimens and prognoses

Among the 14 TW-positive patients, all patients received antibiotic treatment (except for case 2), and the treatment cycle was 6–23 days (median time: 12 days). Fluoroquinolones were used most frequently (71.4%, 10/14), among which the monotherapy or combined regimen of moxifloxacin accounted for 42.9% (6/14). Cephalosporins (cases 5, 11, 12) and macrolides (cases 4, 9) accounted for 21.4% (3/14) and 14.3% (2/14), respectively. In the combined medication strategy, 35.7% (5/14) of the patients received combined antibacterial-antifungal treatment (such as cases 5, 7, 13), and 28.6% (4/14) received combined anti-tuberculosis drugs (cases 1, 6, 9, 14). The efficacy analysis suggested that 85.7% (12/14) of the patients had improved clinical symptoms subsequent to systematic treatment. It is particularly noteworthy that case 7 (a 68-year-old male) received a triple regimen of meropenem-compound sulfamethoxazole-levofloxacin, and the symptoms were relieved after 23 days of treatment. This distinctive phenomenon illustrated that an extended treatment course may be essential for individuals suffering from severe conditions or advanced age. Case 2 (a 17-year-old female) was classified into the non-intervention group, which can be attributed to the fact that she failed to receive drug treatment. Despite the fact that case 13 (a 56-year-old male) received moxifloxacin combined with voriconazole treatment for 11 days, the fatigue symptoms persisted, which may be associated with central nervous system involvement or antibiotic resistance. Under such circumstance, the results require further microbiological verification. In particular, no serious treatment-related adverse reactions were reported in all the improved cases (**Table 4**). The data in this group illustrate that fluoroquinolones (especially moxifloxacin) and combined treatment regimens hold certain clinical value in the management of Whipple’s disease.

**Table 4:** Treatment status and prognosis of 14 patients.

No.	Age	Sex	Clinical manifestation	Antibiotic regimen	Therapy time	Outcome
1	56	Female	Cough, expectoration, abdominal pain, abdominal distension, ascites	Piperacillin sodium, anti-tuberculosis	13	Improved
2	17	Female	/	No medication used	6	Untreated
3	43	Male	/	Amoxicillin	10	Improved
4	38	Male	Fever, cough, expectoration,	Azithromycin	9	Improved
5	17	Male	Cough, expectoration	Ceftazidime for antibacterial, voriconazole for antifungal, etc.	12	Improved
6	56	Male	Cough, expectoration	Levofloxacin, fluconazole, aspirin, anti-tuberculosis	13	Improved
7	68	Male	Cough, expectoration	Meropenem, co-trimoxazole (trimethoprim-sulfamethoxazole), levofloxacin	23	Improved
8	17	Male	/	Moxifloxacin	13	Improved
9	15	Male	Cough, expectoration, wet rales in the lungs	Azithromycin, anti-tuberculosis	10	Improved
10	50	Male	/	Moxifloxacin	22	Improved
11	61	Male	/	Ceftazidime, metronidazole	19	Improved
12	46	Female	Cough, expectoration	Ceftazidime	6	Improved
13	56	Male	Fatigue	Moxifloxacin, voriconazole	11	Uncured
14	45	Male	Cough, expectoration	Moxifloxacin, anti-tuberculosis	14	Improved

## 4 Discussion

As a rare chronic multi-system disorder, Whipple’s disease is characterized by a distinct array of clinical manifestations, encompassing arthritis, persistent diarrhea, significant weight loss, as well as extrapulmonary symptoms like fever, fatigue, and neurological involvement. In recent years, multiple case reports have revealed the potential pathogenicity of TW in respiratory tract infections, especially in individuals enduring immunosuppression or structural lung diseases. Nonetheless, as evidenced by the scarcity of systematic research in this area (Zhang and Xu, 2021; Chen et al., 2024; Li et al., 2024; Shi et al., 2024), a comprehensive understanding remains elusive with regard to the clinical features, imaging findings, and therapeutic approaches tailored to its pulmonary manifestations. When it comes to laboratory diagnosis, *in vitro* culture of TW has always confronted a multitude of significant challenges. TW exhibits insensitivity to conventional culture conditions and sluggish growth, posing challenges for prompt detection through standard microbiological assessments. Despite some literature documenting the successful cultivation and isolation of TW within human macrophages inactivated by Interleukin-4 (IL-4) and human fibroblast cell lines, this culture method approach harbors certain constraints. The average detection and growth time of TW spans an extensive 30 days, rendering it impractical for clinical contexts that necessitate swift diagnosis and intervention (Dolmans et al., 2017). Nonetheless, the clinical diagnosis of TW may be redefined as molecular diagnostic techniques move ahead speedily, especially the application of nanopore sequencing. In 2012, researchers successfully isolated and cultured TW for the first time by utilizing the BALF sample of a patient with acute pulmonary infection. This groundbreaking accomplishment not only reconfirms the presence of TW in pulmonary infections but also underscores the paramount significance of BALF samples in diagnosing pulmonary manifestations of TW (Fenollar et al., 2012).

By utilizing nanopore sequencing technology, this study examined BALF samples and conducted a comprehensive analysis of the clinical characteristics, radiological manifestations, and treatment outcomes of TW-positive patients. As an emerging sequencing technology, nanopore sequencing boasts rapidness, high throughput, and extensive read lengths, thereby enabling more precise detection of TW gene sequences. When compared to mNGS, nanopore sequencing may offer superior advantages in regard to detection speed and accuracy, especially in the detection of low-abundance pathogens in clinical samples (Song et al., 2024b; Zhao et al., 2025a, 2025). For this reason, this study adopted nanopore sequencing technology with the objective of gaining a comprehensive insight into the role of TW in pulmonary infections and digging into its impact on the prognosis of patients. This study is the pioneer in conducting a stratified analysis of patients infected with TW in accordance with the number of sequences obtained by nanopore sequencing (100 vs. ≤ 100). As evidently demonstrated by our research findings, the levels of CRP and LDH in the high-sequence patient group exhibit conspicuously elevated levels, and the difference is statistically significant. This finding suggests that the pathogen load of TW may be tightly correlated with the intensity of the host’s systemic inflammatory response. As an acute-phase reaction protein induced by IL-6, the conspicuous augment in the level of CRP may mirror the activation degree of macrophages by TW (Ngwa et al., 2022). As a facultative intracellular pathogen, TW can escape the degradation of lysosomes and therefore survive for a long time within macrophages, thus continuously releasing pro-inflammatory signals (Ayona et al., 2021; Boumaza et al., 2021; Munjal et al., 2021). Upon activation, macrophages further release a variety of pro-inflammatory cytokines, which subsequently trigger a series of cascading reactions, thereby exacerbating the inflammatory process. Simultaneously, endothelial cells also participate in this process, as they can generate IL-6, which further activates immune cells and drives their involvement in the inflammatory response (Cosenza et al., 2021). This persistent inflammatory provocation could result in the sustained generation of CRP, thereby intensifying the host’s systemic inflammatory response. Prior studies have similarly demonstrated that routine laboratory assessments of individuals with Whipple’s disease often yield nonspecific findings, notably including elevated CRP (Fenollar et al., 2012). On top of that, the increase in the level of LDH may reflect the enhancement of the metabolic activity of immune cells (Gupta, 2022). LDH is an enzyme present in a variety of cells, and its release increases when cells are damaged or metabolically active (Diederichs et al., 1979). As a consequence, the dramatic elevation in the level of LDH may indicate cell damage or metabolic disorders stemmed from TW infection, and it may become a potential biomarker for evaluating the disease activity. As previously demonstrated, the level of LDH is conspicuously increased in individuals suffering from co-infection of TW and other pathogens (Lu et al., 2022). This phenomenon further supports the potential mechanism of action of LDH in TW infection. To put it another way, TW infection may give rise to a conspicuous augment in the release of LDH by activating immune cells and inducing changes in cell metabolism (Lai et al., 2024). It is noteworthy that despite the heightened inflammatory response exhibited by the high-sequence group, no significant disparities were observed in routine infection indicators, including white blood cell count and lymphocyte subsets, between these two groups. This characteristic may result in a limited sensitivity of traditional inflammatory indicators, and CRP and LDH may become more reliable monitoring targets. On that account, it is imperative for future studies to further verify the correlation between the sequence load and clinical severity, as well as to dig into the feasibility of guiding the treatment course through dynamic monitoring of sequence counts.

As revealed by profound studies on the clinical baseline characteristics of TW-positive patients, the proportions of patients in the high-sequence group with clinical manifestations such as fever, cough, and expectoration are higher than those in the low-sequence group. Although these distinctions were not statistically significant, they suggest that the TW sequence counts may be bound up with the clinical characteristics of patients. This may be associated with the pathophysiological mechanism of TW infection. TW infection may trigger the body’s immune response, ultimately bringing about the release of inflammatory factors, thus causing symptoms such as fever, cough, and expectoration. Nevertheless, it remains essential to verify this association by further research. Future studies can not only expand the sample size but also delve deep into the potential mechanism between the TW sequence counts and clinical characteristics. As already demonstrated by imaging studies, the major outlooks of TW pneumonia comprise patchy consolidation, ground-glass opacities, and bronchiectasis, which highly overlap with tuberculosis or nonspecific interstitial pneumonia. This phenomenon thereby gives rise to a comparatively high misdiagnosis rate, which demonstrates consistency with previous research reports (Zhang and Xu, 2021). Nonetheless, some literature has put forth a viewpoint that patients with Whipple’s disease pneumonia exhibit unique radiological manifestations. For example, micronodules can be observed gathering in the upper lungs to form a “galaxy sign”, and the pulmonary infiltrates show a certain degree of mobility before, during, and after treatment, which necessitates further validation by more cases in the future (Li et al., 2024).

With regard to treatment, there is currently no consensus on the treatment regimen for Whipple’s disease. In typical Whipple’s disease, it is recommended to use doxycycline (200 mg/d) and hydroxychloroquine (600 mg/d) for 12 months and then use doxycycline (200 mg/d) for life to avoid reinfection (Lagier et al., 2014). Nonetheless, there is no standard treatment regimen for the special infection type of pulmonary Whipple’s disease. Although moxifloxacin is universally employed in many individuals suffering from Whipple’s disease pneumonia, most of the evidence for its efficacy is on the basis of case reports or small retrospective studies (Lai et al., 2024). Among the 14 TW-positive patients enrolled in this study, the majority of patients received antibiotic treatment, with fluoroquinolones, particularly moxifloxacin monotherapy or combination regimens, which emerged as the most prevalent choice. Efficacy analysis revealed that 85.7% of the patients exhibited clinical improvement post-treatment, underscoring the clinical merit of fluoroquinolones and combined regimens in the management of Whipple’s disease. Nevertheless, the patient’s symptoms in case 7 were relieved after being treated with a triple regimen for 23 days, which also indicates that patients characterized by severe conditions or advanced age may need an extended treatment course. On top of that, although case 13 received combined treatment, the symptoms persisted, which may be bound up with central nervous system involvement or antibiotic resistance.

There are several limitations in the design and implementation of this study. The number of cases included in this case series is less abundant, thereby bringing about a small sample size. This, to some extent, restricts the generalizability and universality of our research findings. The small sample size may also give rise to insufficient statistical power, thereby affecting the ability to detect certain potential associations. In consideration of the above limitations, this study centers around nothing more than the detailed analysis with reference to the clinical features of these cases, aiming to provide foundational data and preliminary insights for subsequent research. With an aim to further validate and expand upon the findings of this study, we will strive to collect more cases, conduct prospective studies, plan to include a negative control group, and carry out multivariate modeling analysis in future research. By increasing the sample size and introducing a control group, we hope to more comprehensively evaluate the clinical features, pathological mechanisms, and potential therapeutic strategies of respiratory diseases caused by Whipple’s disease, thereby providing more instructive research outcomes for clinical practice.

To sum up, pulmonary TW infection, as an emerging clinical entity, necessitates urgent refinement of its diagnostic and therapeutic framework. This study endeavors to systematically reveal the clinical-microbiological association of TW pneumonia, evaluate the prognostic value of the pathogen load, and lay an evidence-based foundation for optimizing the anti-infection strategy by integrating the nanopore sequencing data of BALF, imaging features, and treatment follow-up results. This exploration not only helps to facilitate early recognition of TW infection but also provides a unique model for understanding the interaction between intracellular pathogens and the host.

## 4 Conclusion

Based on the analysis of 14 patients with TW-positive bronchoalveolar lavage fluid via nanopore sequencing, this study demonstrates that TW pneumonia lacks specific imaging features, frequently presenting as patchy shadows, ground-glass opacities, or bronchiectasis, which overlap significantly with tuberculosis or interstitial pneumonia. Higher TW sequence counts (100) were associated with significantly elevated levels of CRP and LDH, indicating a correlation between pathogen load and systemic inflammation. Antibiotic therapy, particularly fluoroquinolones (e.g., moxifloxacin) and combination regimens, showed clinical efficacy, with 85.7% of patients improving symptomatically. However, severe or elderly cases may require extended treatment durations. These findings underscore the importance of nanopore sequencing for accurate diagnosis and support tailored therapeutic strategies based on disease severity and sequence burden.

## Data Availability

The data presented in the study are deposited in the SRA repository, accession number PRJNA1330019.
