# Supplementary material for: Analysis of clinical and imaging features and prognosis of patients positive for Tropheryma whipplei detected by nanopore sequencing of bronchoalveolar lavage fluid
Source: Front Cell Infect Microbiol. 2025 Sep 30;15:1652182. doi: 10.3389/fcimb.2025.1652182 (PMC12518404; doi:10.3389/fcimb.2025.1652182)
Supplement: Supplementary file 1 [file Table1.docx]

Supplementary Material

In this study, the sample pretreatment stage was carried out in a bio-safety cabinet. The operation table was disinfected with 84 disinfectant and 75% alcohol. Subsequently, they were irradiated with an ultraviolet lamp for 30 minutes. Throughout the sample grinding process, the sample was placed in an EP tube. Afterwards, an equal volume of DTT solution, 10 μL of proteinase K (provided by Beijing Puyi Biotechnology Co., Ltd.), 5 μL of lysozyme, and 0.05 mm zirconia grinding beads were added one by one. Then, the sample was ground by adopting a grinder. Notably, the DNA multi-sample library construction auxiliary kit for third-generation nanopore sequencing was used in the nucleic acid extraction stage. Simultaneously, the operation was carried out in line with the kit instructions. The extracted nucleic acid was quality-controlled by Qubit 4.0 to ensure that the nucleic acid concentration and purity meet the requirements of subsequent experiments. Moreover, a reaction system containing 2×PCR mix, specific primers, and a DNA template was prepared with a total volume of 30 μL in the PCR stage. After the PCR reaction was completed, the magnetic bead method was used for purification, and the purified DNA was quality-controlled again by Qubit 4.0. Next, the PCR products were barcode-labeled and the library was constructed. After the library construction was completed in the Nanopore sequencing library construction stage. Concurrently, 100 ng of the library was taken for sequencing on the machine. Afterwards, the GridION platform and MinKNOW software were used to collect real-time sequencing data. In the data analysis stage, quality filtering was first performed on the original sequencing reads, and fragments smaller than 200 bp were removed. Subsequent to the above steps, host DNA reads were removed by aligning with the human reference genome. The remaining reads were then aligned with the pathogenic microorganism database and the drug resistance gene database, so as to generate the analysis results of pathogenic microorganism species and drug resistance genes. Throughout the entire process, the dosage of all reagents and the operation steps were strictly carried out in accordance with the instructions of each kit and the technical guidelines provided by the company to ensure the accuracy and reliability of the experimental results.
